# Achalasia associated with two epiphrenic diverticula

**DOI:** 10.1590/0100-3984.2015.0019

**Published:** 2016

**Authors:** Stéphano Santos Belisário, Gabriel Antonio de Oliveira, Rodrigo Stênio Moll de Souza, Thais Julio Pacheco, Elton Francisco Pavan Batista

**Affiliations:** 1 Universidade Federal do Espírito Santo (UFES), Vitória, ES, Brazil.

**Figure 1:**
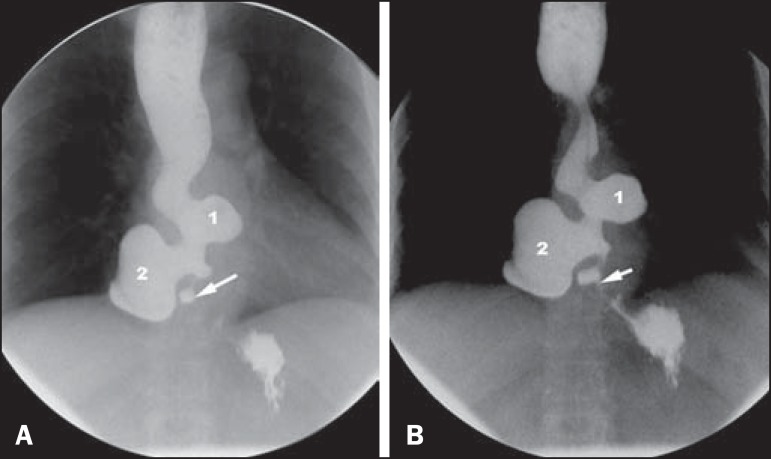
Barium swallow image sequences, obtained with the patient standing, showing narrowing of the lumen and spasm of the distal esophagus, indicating achalasia (arrow). Immediately above, two diverticula can be seen (1 and 2). The proximal esophagus is dilated and shows impaired peristalsis.

**Figure 2:**
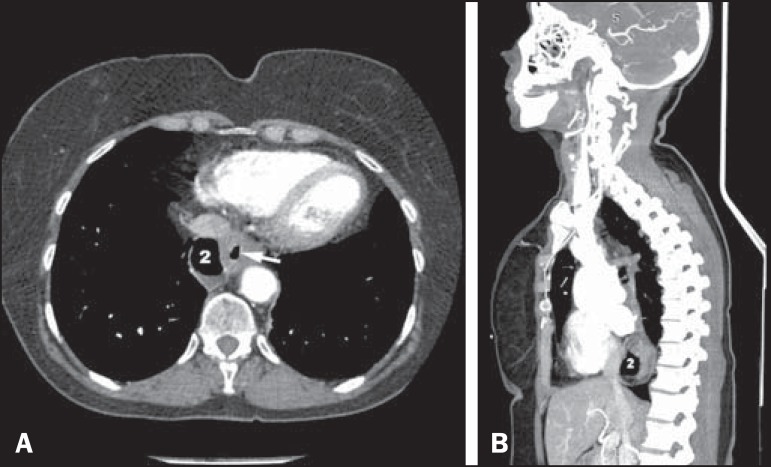
**A:** Axial CT slice, with a mediastinal window, demonstrating air in diverticulum 2 and in the esophageal lumen (arrow). **B:** Reformatting in the sagittal plane, showing the posterior position of diverticulum 2.

Dear Editor,

Here, we present the case of a 63-year-old, previously healthy, female patient who sought treatment at a general surgery outpatient clinic complaining of an approximate one-year history of dysphagia for solids. An upper gastrointestinal series showed achalasia and two diverticula in the distal esophagus (Figure 1). Those findings were also documented by computed tomography (Figure 2) and upper gastrointestinal endoscopy. The latter identified an area of esophagitis in one of the diverticula, and that was confirmed by biopsy.

Diverticula that occur in the distal 10 cm of the esophagus, known as epiphrenic diverticula, can be congenital or acquired. The congenital form, which is extremely rare, results from communication between the esophageal lumen and a duplication cyst. Those are true diverticula, with mucosa, submucosa, a muscle layer, and adventitia. Acquired diverticula are actually pseudodiverticula, formed by herniation of the mucosa and submucosa through the muscle layer. Such herniation is caused by increased pressure in the esophageal lumen. Therefore, acquired diverticula are referred to as traction pseudodiverticula. There are always predisposing conditions, such as collagen diseases, hiatal hernias, and, especially, esophageal motility disorders^(1,2)^.

The best imaging method for the initial approach to esophageal disorders is an upper gastrointestinal series, because it is noninvasive and can demonstrate not only the anatomy but also esophageal motility^(1)^.

Dysphagia for solids, the main complaint of the patient, is a nonspecific symptom and can occur in various esophageal disorders. Many epiphrenic diverticula are asymptomatic or only mildly symptomatic^(3)^. When present, symptoms generally arise from impaired peristalsis. In the case presented here, the symptoms were probably caused by the achalasia. The occurrence of two epiphrenic diverticula in the same patient, as in this case, is rare^(1,4)^.

The retention of residues in diverticula can cause halitosis, regurgitation, aspiration pneumonia, and esophagitis^(4)^. The condition can evolve to metaplasia of the epithelium, which would explain the increased risk of developing esophageal cancer (as occurs in 0.3-3.0% of cases). Episodes of gastrointestinal bleeding can also occur^(3)^.
